# The Effect of Chronic Inflammation and Oxidative Stress on Alzheimer's Disease Progression: A Systematic Review

**DOI:** 10.7759/cureus.84057

**Published:** 2025-05-13

**Authors:** Elisa A Bornemann, Hari Krishna Kamma, Mohammad Alabbas, Mohammad Elashahab, Naushad Abid, Sara Manaye, Kaaviya Cheran, Chinmayee Murthy, Sheiniz Giva, Sai Sri Penumetcha

**Affiliations:** 1 Neurology, California Institute of Behavioral Neurosciences and Psychology, Fairfield, USA; 2 College of Medicine, California Institute of Behavioral Neurosciences and Psychology, Fairfield, USA; 3 Cardiology, University of Debrecen, Debrecen, HUN; 4 Radiology, California Institute of Behavioral Neurosciences and Psychology, Fairfield, USA; 5 Rheumatology, King Faisal University, Al-Ahsa, SAU; 6 Medicine, California Institute of Behavioral Neurosciences and Psychology, Fairfield, USA; 7 General Medicine, California Institute of Behavioral Neurosciences and Psychology, Fairfield, USA; 8 Internal Medicine, California Institute of Behavioral Neurosciences and Psychology, Fairfield, USA; 9 Neonatology, Temple University Hospital, Dublin, IRL; 10 General Medicine, Chalmeda Anand Rao Institute of Medical Sciences, Karimnagar, IND

**Keywords:** alzheimer's disease, metabolic syndrome, neuroinflammation, oxidative stress, pro-inflammatory state., systemic inflammation

## Abstract

Alzheimer’s Disease (AD) is known to be the most common type of dementia among older adults. It is characterized by a gradual decline in cognitive abilities, particularly the deterioration of short-term memory. The hallmark neuropathology of AD is the accumulation of neurofibrillary tau tangles (NFTs), which consist of hyperphosphorylated tau protein, as well as extracellular beta-amyloid plaques in the brain. Evidence suggests that AD is not solely tied to neurological mechanisms, and that other factors, such as inflammation, can affect disease progression, including systemic inflammation seen in metabolic syndrome and oxidative stress. We performed a literature review by searching databases and conducting a manual search of studies regarding the relationship between AD, inflammation, and oxidative stress, following the Preferred Reporting Items for Systematic Reviews and Meta-Analyses (PRISMA) guidelines. After meticulous scrutiny and the application of inclusion and exclusion criteria to clinically relevant papers, 14 studies were deemed relevant for this review regarding the effect of inflammation and oxidative stress in relation to AD and its progression. The findings conclude that there is new evidence supporting the theory that chronic inflammation plays a significant role in the progression of the disease, which could allow for future advancements in treatments, diagnostics, and preventive tools for its management. These advancements could include the implementation and use of biomarkers for inflammation, the use of algorithms to stratify the disease’s grade, and the use of mineral supplements like zinc. Furthermore, the management of underlying conditions has been shown to be beneficial in slowing the progression of AD.

## Introduction and background

Alzheimer's Disease (AD) is known to be the most common type of dementia. According to studies, the number of AD cases has exponentially increased with the aging global population, and the number of AD patients is expected to rise, reaching 66 million by 2030 and 115 million by 2050. By 2050, it is estimated that more than 70% of dementia patients will reside in low- and middle-income nations, where the increase will be the greatest [1].

AD is a neurodegenerative disorder characterized by progressive loss of cognitive functions, in particular, short-term memory loss [2]. The causatives of AD are not yet fully known, but the probabilities are a combination of genetic, environmental, and lifestyle factors [3]. The neuropathology hallmarks of AD-suffering brains are characterized by extracellular accumulation of beta-amyloid (Aβ) plaques (Ab40 and Ab42) and intraneuronal neurofibrillary tau tangles (NFTs) deposition composed of hyperphosphorylated tau protein (p-tau) [3,4]. Furthermore, the role of microglia involvement and conversion into a pro-inflammatory phenotype after interaction with b-amyloid plaques and its decreased incidence when anti-inflammatory measures are taken supports the role of the microglia pathophysiology and possible target of anti-inflammatory therapies [5,6].

While different theories prevail in the medical community regarding the causes of developing AD, some suggest the further involvement of inflammation and its elevated biomarker presence in AD cases [6]. Nevertheless, many of these studies have not been able to conclude the veracity of the possible loss of regulation of the pro-inflammatory response to be linked to the development of the disease. 

Some theories suggest that activated microglia can release a variety of cytokines and are thought to have the ability to cause chronic neuroinflammation, as seen in AD. This persistent immunological response in the brain has been seen in AD and other neurological pathologies [7].

The most common topic of research on AD inflammation has been cytokines, but there have also been studies on complement, chemokines, growth factors, oxidative stress, microglial activation, astrocyte reactivity, and other subjects. In actuality, however, inflammatory mechanisms are highly interactive and hardly ever take place independently of one another [8]. This review aims to summarize and inform readers about some of the available investigations and studies being conducted on systematic inflammation and its connection to AD and its progression. 

## Review

Methods

We developed this systematic review in agreement with the Preferred Reporting Items for Systematic Reviews and Meta-Analyses (PRISMA) (Page MJ, et al., 2020) [9]. The databases navigated for the development of this review were: PubMed, PubMed Central, Medline, and Google Scholar.

Keywords

The following keywords were used to perform the search: Alzheimer's disease, systemic inflammation, oxidative stress, neuroinflammation, metabolic syndrome, and proinflammatory state. A manual search was also done by looking for relevant review articles. 

Search Strategy

The final search strategy includes keywords and Medical Subject Headings (MeSH). The final search strategy used for PubMed database (PubMed, PubMed Central, and Medline) and Google Scholar was: Oxidative stress ( "Oxidative Stress/drug effects"[Majr] OR "Oxidative Stress/etiology"[Majr] OR "Oxidative Stress/radiation effects"[Majr] ) OR metabolic syndrome ( "Metabolic Syndrome/analysis"[Majr] OR "Metabolic Syndrome/blood"[Majr] OR "Metabolic Syndrome/chemically induced"[Majr] OR "Metabolic Syndrome/complications"[Majr] OR "Metabolic Syndrome/etiology"[Majr] OR "Metabolic Syndrome/metabolism"[Majr] OR "Metabolic Syndrome/physiopathology"[Majr] ) AND Alzheimer disease OR dementia OR senile dementia OR Alzheimer dementia OR ( "Alzheimer Disease/analysis"[Majr] OR "Alzheimer Disease/chemically induced"[Majr] OR "Alzheimer Disease/congenital"[Majr] OR "Alzheimer Disease/diet therapy"[Majr] OR "Alzheimer Disease/epidemiology"[Majr] OR "Alzheimer Disease/etiology"[Majr] OR "Alzheimer Disease/metabolism"[Majr] ) AND Progression. A manual search was also done by looking for relevant review articles.

Inclusion and Exclusion Criteria

Studies published between the years 2018 and 2022 were included. Article types: abstracts, free full-text, and full-text were part of the inclusion criteria. Articles that discussed studies made on the human species in the age group of more than 65 and both gender groups were all part of the inclusion criteria. Non-English literature and younger age groups were excluded.

Screening and Quality Assessment 

The authors screened the titles and abstracts of the relevant results, and the final selected articles were assessed depending on the contents of each article using quality assessment tools to assess them individually. The quality assessment tools used were Cochrane to asses randomized trial articles; each qualifying article was assessed and summarized the risk of bias (low or high risk) across all domains of this tool to conclude the overall risk of bias and how it might impact the study. New Castle Ottawa Tool assessed the quality of nonrandomized studies, including case-control and cohort studies a score of more than seven was considered a low risk of bias and, hence, of high quality, and a score of less than three was considered a high risk of bias and low quality, A Measurement Tool to Assess Systematic Reviews (AMSTAR) (systematic reviews articles), and Scale for the Assessment of Narrative Review Articles (SANRA) checklist was used to assess the quality of narrative review articles and other research articles without clear method section, articles that satisfied more than 60% of the checklist were considered high quality.

The inclusion criteria are illustrated in Table 1.

**Table 1:** Inclusion criteria applied to filter articles AMSTAR: A Measurement Tool to Assess Systematic Reviews; SANRA: Scale for the assessment of non-systematic review articles

Years since publication	Type of study	Language	Type of subjects	Age of subjects	Quality assessment tool
5 years between 2018-2022	Abstracts, free full text, and full text	English language	Studies were made in humans, male and female	more than 65 years of age	Cochrane, New Castle Ottawa Tool, AMSTAR, and SANRA

Results

Included Studies

Our literature search yielded a total of 2,452 articles, 2,438 articles from PubMed, PubMed Central, and Medline using MeSH search, and 14 articles from a Google Scholar search done by manual search. Cero duplicates were found. 

The 2,452 articles were manually screened by title, followed by 43 articles manually screened by abstract. The remaining 38 articles were assessed for eligibility using quality assessment tools depending on the article content. The tools used for eligibility were: Cochrane (randomized trials articles), New Castle-Ottawa Tool (nonrandomized studies, including case-control and cohort studies), AMSTAR (systematic reviews articles), and SANRA (other research papers without a clear method section). Of these 38 articles, 20 satisfied the criteria and quality standards to be further included in the review. Out of the 20 selected articles, 13 are Cohort studies, two are randomized controlled trials, and five are reviews.

**Figure 1:**
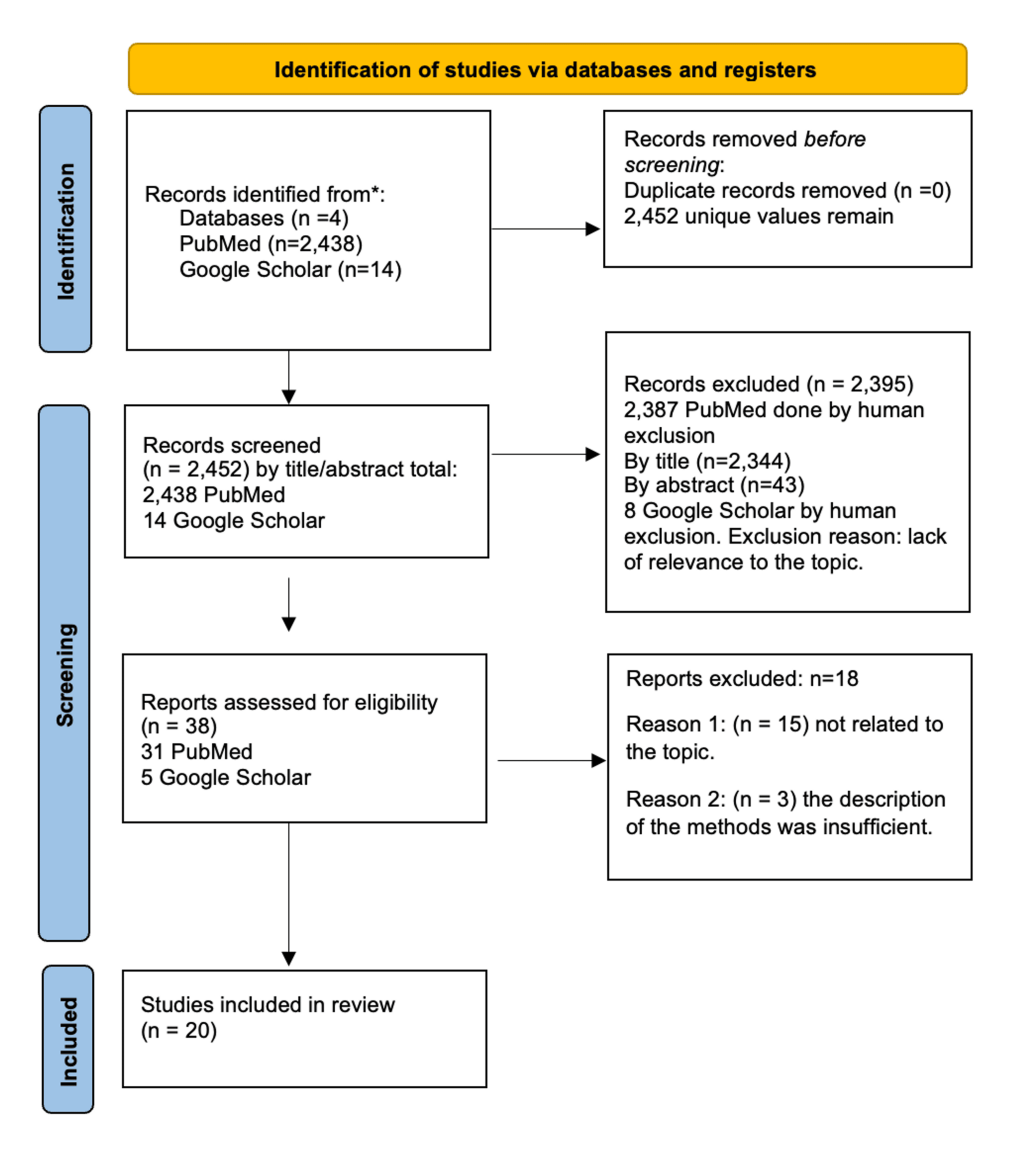
PRISMA flow diagram with a description of the sort process for article selections ^*^Reported numbers are from each database and register searched (rather than the total number across all databases and registers). PRISMA: Preferred Reporting Items for Systematic Reviews and Meta-Analyses

We outline the screening process for article selection in the PRISMA flow diagram in Figure 1 below. 

In the selected studies, most of them analyzed the progression of the disease in different groups to fulfill the purpose of each study. Many focused on Mild Cognitive Impairment (MCI) and AD patients, with results compared to healthy control groups (CTL). 

Some studies were able to make strong prognoses based on the predictability of disease development and progression, using methods of differentiation and prediction of its longitudinal course, along with biomarker concentrations and imaging correlations, as the disease progresses from the prodromal to the illness stage. Other studies implemented the relationship of certain metabolites and their concentrations to the progression of AD.

Discussion

This systematic review focuses on studies that analyzed biomarkers of neuroinflammation (n=3, Table 2), projecting the progression of the disease and their concentrations; pathophysiological changes in AD (n=5, Table 3); and studies on the role of metabolites in reducing the progression of AD (n=6, Table 4).

**Table 2:** Summary of the selected studies on biomarkers of neuroinflammation AD: Alzheimer’s disease; MCI: mild cognitive impairment; CTL: control group; CSF: cerebral spinal fluid; CER: ceruloplasmin; CRP: C-reactive protein; UA: uric acid; Hcy: homocysteine

Author and publication	Purpose of the study	Number of participants in the study	Intervention studied	Results/conclusions
Janelidze S et al., 2018 [10]	Measure CSF reflecting neuroinflammation and cerebrovascular changes, and study their association with AD markers, imaging correlates, and the clinical progression of the disease over time	1,014 subjects divided in cognitively normal CTL (n=508), mild cognitive complaints (n=449), and patients with AD dementia (n=57)	CSF concentration of neuroinflammatory and cerebrovascular biomarkers	Neuroinflammation and cerebrovascular dysfunction are early events that occur at the presymptomatic stages of AD and contribute to disease progression
Morgan et al., 2019 [11]	Studies of inflammatory biomarkers and complement proteins differ in neurologically normal elderly individuals (CTL), MCI, and AD, as well as the relationship between them	A total of 720 subjects: 262 patients with AD, 199 patients with MCI, and 259 CTL patients	Plasma analysis of 53 different proteins relevant to inflammation	Ten of the 53 proteins showed significance in the study: complement components (C3, C4, C5), two complement regulators (FH, FI), a soluble form of a complement receptor (sCR1), a classical marker of inflammation (CRP), and three chemokines (eotaxin-1, MCP-1, and MIP-1b), demonstrating high predictability in models comparing AD to CTL
Pu, et al., 2017 [12]	Study the oxidative stress markers and metal ions in AD patients	One hundred twenty-five participants with AD, including mild AD (n=28), moderate AD (n=42), and severe AD (n=55), as well as CTL (n=40)	Serum levels of CER, CRP, UA, Hcy, copper, iron, and zinc	Decreased levels of serum CER, UA, and zinc and increased levels of serum copper can be possible risk factors of severity of AD

**Table 3:** Summary of the selected inflammasome effects and their impact on AD progression AD: Alzheimer’s disease; OS: oxidative stress; MCI: mild cognitive impairment; aMCIA: amnestic mild cognitive impairment; PBMCs: peripheral blood mononuclear cells; CSF: cerebral spinal fluid; LPS: lipopolysaccharides; GSDMD Gasdermin D; PET: positron emission tomography; ApoE: apolipoprotein E; NDAN: nondemented with Alzheimer’s neuropathology; CRP: C-reactive protein

Author and publication	Purpose of the study	Number of participants in the study	Intervention studied	Results/conclusions
Ismail R, et al., 2020 [2]	To assess via PET the relationship between levels of inflammation and the loads of aggregated β-amyloid and tau at baseline and after two years in the prodromal AD	43 subjects with MCI	PET samples: serial C-PK11195 PET, C-PiB PET, and F-Flortaucipir PET	There is a correlation between microglial activation and the rise in β-amyloid load, followed by a decline in microglial activation as the β-amyloid load approaches AD levels
Cullen NC, et al., 2021 [13]	To find the relationship between inflammatory protein levels and their association with the chronological brain age of healthy individuals without AD and patients with an AD diagnosis	859 individuals	CSF and plasma samples: 73 inflammatory proteins were measured in both CSF and plasma	Inflammatory aging can be measured without hesitation in AD and is related to pathophysiological changes during the progression of the disease
Fracassi A, et al., 2021 [14]	To study the relationship between amyloid overload, OS, and the cellular response	34 specimen samples: 12 Ctrl, 10 AD, and 12 NDAN	Brain tissue derived from human autopsy	Shows new evidence that confirms the role of redox imbalance in the pathogenesis of AD and the importance of the antioxidant protective effect in coping with Aβ-mediated disease and its progression
Rui W, et al., 2021 [15]	To examine the relationship between systemic inflammasome-induced pyroptosis and clinical features in aMCI and AD	A total of 86 participants: 33 with aMCI, 33 with AD, and 20 cognitive normal controls	Peripheral blood samples: levels of inflammasome-related genes and proteins in PBMCs, as well as levels of IL-1β, Aβ1-42, Aβ1-40, p-tau, and t-tau in CSF and plasma IL-1β levels. LPS levels in mouse models	Strong evidence suggests that inflammasome signaling and GSDMS-induced pyroptosis are activated in PBMCs of aMCI and AD patients. Additionally, the proinflammatory cytokine IL-1β has been demonstrated to have a strong association with the pathophysiology of aMCI and AD
Tao Q, et al., 2018 [16]	To study interaction between ApoE and chronic low-grade inflammation and its incidence of AD	Final study included 2,656 human participants	Peripheral blood sample: serum CRP, dementia diagnosis including AD and others, and brain volume	Low-grade inflammation in ApoE4 was associated with a shortened latency of AD onset

**Table 4:** Studies on therapeutic treatments CSF: cerebral spinal fluid; INI: intranasal insulin; AD: Alzheimer’s disease; CT: control subjects; FAAH: fatty acid amide hydrolase; eCB: endocannabinoid system; MCI: mild cognitive impairment; ML: machine learning; CSF: cerebral spinal fluid; sTREM2: soluble trigger receptor expressed on myeloid cells 2; IL: interleukin; MetS: metabolic syndrome; PBMC: peripheral blood mononuclear cell

Author and publication	Purpose of the study	Number of participants in the study	Intervention studied	Results/conclusions
Kellar D, et al., 2022 [17]	Assess the effect of INI on CSF markers of inflammation, immune function, and vascular function, and their relationship with the progression of AD	49 participants enrolled in the study	Imaging, CSF, inflammation, immune, and vascular markers.	INI treatment can modulate markers of immune function, inflammation, and vascular integrity, and may suggest the activation of a compensatory immune response
Chiurchiù, et al., 2021 [18]	Potential relationship of the eCB system in modulating immune cell populations in the peripheral blood of AD patients	30 participants: 15 CNT and 15 AD patients	PBMC samples	Peripheral immune cells, specifically FAAH, have been shown to play an important role in regulating AD monocytes/macrophages
Huang YL, et al., 2021 [19]	Characterize MCI and AD plasma metabolites in untargeted metabolomic profiles	61 human plasma samples, and 48 MCI diagnosis patients	Human plasma samples of metabolic signatures	With the help of ML algorithms and a combination of untargeted metabolomics, an evaluation will be provided to assist in the stratification of patients with MCI
Rivers-Auty J, et al., 2021 [20]	Zinc status can influence the development of AD	1,631 human adults were recruited, and animal model experiments were conducted	Plasma blood sample of zinc value of both subject models (human and animal)	Zinc supplementation is associated with slower cognitive decline and a lower prevalence of AD, while subclinical zinc deficiency accelerates memory decline in animal models
Jensen CS, et al., 2019 [21]	Demonstrate the upregulating effect of physical activity on anti-inflammatory cytokines	198 patients with AD	Plasma and CSF levels of 8-isoprostane, sTREM2, and the MSD V-PLEX Proinflammation Panel 1 in humans, containing interferon gamma, IL-10, IL-12p70, IL-13, IL-1β, IL-2, IL-4, IL-6, IL-8, and tumor necrosis factor alpha	Most inflammatory markers did not change after exercise, except for sTREM2, suggesting a small systemic inflammatory effect related to physical activity in patients with AD
Fan YC, et al., 2017 [22]	To evaluate the association between changes in metabolic syndrome status and the incidence of developing dementia	3,458 participants between the ages of 40 and 80 years	Based on diagnosis of MetS, participants were classified in three groups: persistent, nonpersistent, and non-MetS	In this 10-year follow-up study, it was demonstrated that patients with worsened metabolic syndrome (persistent MetS) had an increased risk of developing dementia

The data collected from the included studies are organized in Tables 2, 3, 4, summarizing the purpose of each study, the number of participants, the intervention studied, and a brief conclusion for each of the selected studies. Also, the studies are organized based on their published date in chronological order, from newer to older studies. 

Biomarkers of Neuroinflammation

There have been many studies suggesting the relationship between neuroinflammatory biomarkers and other proteins relevant to inflammation that suggest the possibility of being related to the progression of AD [8]. For the purpose of this review, we have included three studies in Table 2, where markers of neuroinflammation were analyzed and their correlation to the progression of AD.

In the selected studies, we can appreciate different methods of intervention (plasma analysis and cerebrospinal fluid (CSF)) along with different markers, all related to systemic, neurological inflammation, and potential antioxidant components used to decrease or increment inflammation in the human body. 

In the study of CSF markers related to neuroinflammation and cerebrovascular changes, data were analyzed from various associations correlating CSF biomarkers (YKL-40, interleukin (IL)-6, IL-7, IL-8, IL-15, interferon-g-induced protein 10 (IP-10), monocyte chemoattractant protein 1 (MCP-1), intercellular adhesion molecule 1 (ICAM-1), vascular adhesion molecule 1 (VCAM-1), placental growth factor (PIGF), and FMS-related tyrosine kinase 1 (Flt-1)) with amyloid pathology, tau, gray matter atrophy, and AD progression. The results showed that levels of chitinase-3-like protein 1 (YKL-40), ICAM-1, VCAM-1, IL-15, and Flt-1 were increased in AD during the preclinical and prodromal stages, particularly in CSF tau and Ab-positive individuals, with especially high levels of YKL-40 showing the strongest association in Ab-positive individuals. Additionally, high levels of these biomarkers were associated with cortical thinning, as identified in this study [10].

Based on these studies, the results show significant predictability in the progression of AD and some systemic inflammatory markers such as high serum complement components (C3, C4, C5), complement regulator proteins (FH, FI), a soluble form of a complement receptor (sCR1), a classical marker of inflammation (CRP), and chemokines (eotaxin-1, MCP-1, and MIP-1b) [11].

Lastly, we analyzed the correlation between plasma biomarker levels of oxidative stress, including ceruloplasmin (CER), C-reactive protein (CRP), uric acid (UA), and homocysteine (Hcy), and serum metal ion levels, including copper, iron, and zinc, in relation to disease severity in AD patients at varying stages of progression. The results of the study conclude that there is a significant correlation between the severity of dementia, especially moderate to severe dementia, and serum CER, UA, copper, and zinc, as well as increased levels of serum copper as a possible determinant risk factor of the severity progression of AD [12].

In this context, these three studies propose a strong relationship and demonstrate that biomarkers in plasma and CSF predict the link between their presence and the progression of AD, potentially providing future targets for the treatment and prevention of the disease, while considering certain factors. Additionally, this could suggest possible alternatives for diagnosing the disease and allow projections of its progression over time.

Inflammasome Effects in the Progression of AD

Of the selected studies, the intervention characteristics used were radiographic studies (PET), blood plasma and CSF, and pathology brain specimens [2,13-16]. Although the studies had different interventions, they all shared the common goal of finding a relationship between inflammation and the progression of AD.

The relationship between chronic inflammation and its possible effects on the neuronal system is a well-studied field, which has been supported by many other studies throughout time, proving its strong relationship and possible correlation. Nevertheless, there are many gaps still today, and we aim to summarize more knowledge from some of the new studies. 

To accomplish the purpose of this review, we have included in Table 3 recent studies that provide a strong medical background on the correlation between the inflammatory process and the progression of AD.

Inflammation has been shown in past studies to be highly related to AD progression, and the implications of its progression [13]. The presence and elevation of inflammatory proteins in CSF and plasma will correlate to the value of amyloid-b(Ab) positive and cognitively impaired subjects (Ab-CU) building on past data establishing the enrolment of innate immune system changes in healthy and impaired patients, under this premise, this study shows the accelerated aging of the immune system in MCI and AD patients; the results of this study can be used in medical practice to identify and track the progression of AD [13]. 

On the other hand, a viable study of inflammation is the measure of inflammasomes and proinflammatory cytokines such as IL, especially IL-1β, which has been linked to neuroinflammation. To predict if there is a relationship between peripheral inflammasomes and dementia, levels of protein in peripheral blood mononuclear cells (PBMCs) were studied. Studies on Gasdermin D (GSDMD) have followed since it was previously linked to pyroptosis and the release of IL-1β. The results showed a significant increase in GSDMD expression in MCI and AD patients, and subsequently, IL-1β levels were higher in plasma and CSF in MCI and AD patients than in the CNT group. This study concludes that this could potentially indicate future methods for treating pyroptosis brought on by inflammasomes [15]. 

Another question of interest that scientists raised and that we found to be useful for the purpose of this review is the interaction between low-grade chronic inflammation and apolipoprotein E (ApoE) genotype and its association with the development of AD. In this study, Tao Q et al. measured the genotype and concentrations of ApoE, C-reactive protein (CRP), a biomarker for low-grade inflammation, and the risk of AD. Brain magnetic resonance imaging was also performed to support secondary analyses. During the development of this study, various associations were made, with the most significant being the strong link between the ApoE4 allele and chronic low-grade inflammation (as indicated by CRP measures), which was strongly associated with an increased risk of AD and dementia. The clinical relevance of this study lies in providing preliminary evidence of the significance of the ApoE genotype in studies of anti-inflammatory treatments for AD in future clinical trials [16]. 

Of the other studies, the intervention studied differed from the rest, where Fracassi A, et al. used brain tissue derived from human autopsies [2,14]. This study’s goal was to look at the connections between amyloid excess, oxidative stress, and cellular response brought on by AD. This study concluded that redox imbalances play a critical role in the pathogenesis of AD, highlighting the necessity of strong antioxidant defenses to handle AD-mediated damage and the significance of the antioxidant protective action in preventing the advancement of Aβ-mediated illness [14]. Lastly, we review a study in which patients with MCI underwent serial C-PK11195 positron emission tomography (PET) scans over a period of two years, with the hypothesis that levels of inflammation and aggregated β-amyloid and tau concentrations would differ between the prodromal and illness stages of the disease. Concluding that over a period of two years, microglial activation and increasing amyloid load are correlated, with the following decline of microglial activation as amyloid load reaches AD levels [2]. 

Therapeutic Treatments 

In Table 4, we present a compilation of recent treatment experiments that suggest preventive protocols to consider for aging patients at risk of developing MCI and AD. Most of the studies selected considered measuring plasma blood samples, while others considered CSF and image-related correlations. The following are some conclusions of new studies of therapeutics in the intent of prevention, diagnostics, and therapeutics in the management of AD. These studies can be considered preventive actions for older patients. Fan et al. present in this large cohort the increased risk of developing dementia in patients who have experienced a worsening of MetS over time [22]. 

Zinc deficiency has been proven in animal models to be a determining factor in the incidence of dementia via enhancing the inflammatory reaction carried out by the NLRP3 inflammasome [20]. The conclusion of this study comes after looking at the significantly lower prevalence of AD in patients taking supplements. The six most popular mineral supplements studied were: calcium, iron, magnesium, multivitamins, selenium, and zinc, discovering that taking calcium, iron, magnesium, and zinc supplements lowered the prevalence of AD more than not taking the supplements [20]. 

Lastly, the effect of using physical activity as a potential anti-inflammatory agent in patients suffering from AD, physical exercise after 16 weeks showed to have an effect on markers of neuroinflammation. Furthermore, physical activity may have a slight systemic inflammatory effect in AD patients [21]. 

The following study specifically looked at how intranasal insulin (INI) affected CSF indicators of inflammation, immunological function, and vascular function, as well as how these markers were related to clinical markers of AD development. As a result, findings demonstrated that INI therapy altered immune function, inflammation, and vascular integrity markers, supporting activation of other immune response mechanisms, suggesting that insulin may alter the progression of AD [17]. 

Other studies based on other possible therapeutic targets suggest the use of regulating AD monocytes/macrophages and the role of fatty acid amide hydrolase [18]. This study was based on the analysis of the endocannabinoid (eCB) system, and the measure of elements of such system concluding that by decreasing the production of pro-inflammatory cytokines like TNF-, IL6, and IL-12 and increasing the production of the anti-inflammatory cytokine IL-10 in monocytes and monocyte-derived macrophages from AD patients, pharmacological FAAH inactivation was able to modulate their immune response [18].

The last study that we are going to cover in this review is based on metabolites profiling with the help of machine learning technologies, the 20 metabolites studied for the purpose of this research were connected to the development and existence of AD in patients, a metabolite panel is established in the study to help in the future classification of MCI patients to forecast the conversion to AD [19].

The goal of this systematic review was to learn about new therapeutic investigations that are being made in relation to systemic inflammation and its connection to AD and its progression. We were glad to find very diverse approaches in the study of AD, developing more strong knowledge to understand the etiology of the disease and, from there, developing new therapeutics and preventive actions [13-19]. Long longitudinal studies have shown to demonstrate richer bases for the progression of the disease, and we were glad to find this type of study in our research [10,16,19,20,22]. 

Limitations 

This article faces several limitations identified through its development, some of which are: population over 65 years old; no studies of the younger population, with or without a known predisposition to the illness, were included; male-prevalent studies, as most of the patients enrolled in these studies are male, and female-only studies were not commonly found. Additionally, to keep this research up-to-date, studies older than five years after publication were excluded from this review, missing significant and potentially relevant investigations conducted in the past.

## Conclusions

This systematic review examined current evidence on the roles of oxidative stress and systemic inflammation in the pathogenesis and progression of AD. Although chronic inflammation has been consistently associated with AD progression, the precise mechanisms underlying this relationship remain incompletely understood. Nevertheless, the growing body of literature reveals promising directions for both mechanistic insights and therapeutic development. The studies reviewed highlighted the potential of pro-inflammatory biomarkers, detectable in plasma or cerebrospinal fluid, as tools for monitoring disease progression and informing clinical decision-making. Additionally, emerging research supports the application of machine learning techniques in metabolomics to facilitate patient stratification and advance precision medicine approaches in AD care.

The role of oxidative stress was also reinforced through multiple high-quality studies, which demonstrated that redox imbalance contributes significantly to neurodegeneration. Evidence suggests that antioxidant interventions, including micronutrient support (e.g., zinc), regular physical activity, and well-managed control of comorbidities such as metabolic syndrome, may have protective effects and contribute to symptom reduction in at-risk populations. Despite these advances, definitive conclusions regarding the causal roles of the correlation between inflammation and oxidative stress in Alzheimer's disease remain elusive. Continued investigation, particularly through longitudinal and integrative research designs, is essential to clarify these complex biological processes and to translate emerging findings into effective therapeutic strategies. Ultimately, such efforts are critical to slowing disease progression and improving outcomes for individuals affected by AD.
